# Material radiopurity control in the XENONnT experiment

**DOI:** 10.1140/epjc/s10052-022-10345-6

**Published:** 2022-07-08

**Authors:** E. Aprile, K. Abe, F. Agostini, S. Ahmed Maouloud, M. Alfonsi, L. Althueser, E. Angelino, J. R. Angevaare, V. C. Antochi, D. Antón Martin, F. Arneodo, L. Baudis, A. L. Baxter, L. Bellagamba, R. Biondi, A. Bismark, A. Brown, S. Bruenner, G. Bruno, R. Budnik, C. Capelli, J. M. R. Cardoso, D. Cichon, B. Cimmino, M. Clark, A. P. Colijn, J. Conrad, J. J. Cuenca-García, J. P. Cussonneau, V. D’Andrea, M. P. Decowski, P. Di Gangi, S. Di Pede, A. Di Giovanni, R. Di Stefano, S. Diglio, A. Elykov, S. Farrell, A. D. Ferella, H. Fischer, W. Fulgione, P. Gaemers, R. Gaior, M. Galloway, F. Gao, R. Glade-Beucke, L. Grandi, J. Grigat, A. Higuera, C. Hils, K. Hiraide, L. Hoetzsch, J. Howlett, M. Iacovacci, Y. Itow, J. Jakob, F. Joerg, N. Kato, P. Kavrigin, S. Kazama, M. Kobayashi, G. Koltman, A. Kopec, H. Landsman, R. F. Lang, L. Levinson, I. Li, S. Liang, S. Lindemann, M. Lindner, K. Liu, F. Lombardi, J. Long, J. A. M. Lopes, Y. Ma, C. Macolino, J. Mahlstedt, A. Mancuso, L. Manenti, A. Manfredini, F. Marignetti, T. Marrodán Undagoitia, K. Martens, J. Masbou, D. Masson, E. Masson, S. Mastroianni, M. Messina, K. Miuchi, K. Mizukoshi, A. Molinario, S. Moriyama, K. Morå, Y. Mosbacher, M. Murra, K. Ni, U. Oberlack, J. Palacio, R. Peres, J. Pienaar, M. Pierre, V. Pizzella, G. Plante, J. Qi, J. Qin, D. Ramírez García, S. Reichard, A. Rocchetti, N. Rupp, L. Sanchez, J. M. F. dos Santos, G. Sartorelli, J. Schreiner, D. Schulte, H. Schulze Eißing, M. Schumann, L. Scotto Lavina, M. Selvi, F. Semeria, P. Shagin, E. Shockley, M. Silva, H. Simgen, A. Takeda, P. L. Tan, A. Terliuk, C. Therreau, D. Thers, F. Toschi, G. Trinchero, C. Tunnell, F. Tönnies, K. Valerius, G. Volta, Y. Wei, C. Weinheimer, M. Weiss, D. Wenz, J. Westermann, C. Wittweg, T. Wolf, Z. Xu, M. Yamashita, L. Yang, J. Ye, L. Yuan, G. Zavattini, Y. Zhang, M. Zhong, T. Zhu, J. P. Zopounidis, XENON Collaboration, M. Laubenstein, S. Nisi

**Affiliations:** 1 Physics Department, Columbia University, New York, NY 10027 USA; 2 Kamioka Observatory, Institute for Cosmic Ray Research, and Kavli Institute for the Physics and Mathematics of the Universe (WPI), University of Tokyo, Higashi-Mozumi, Kamioka Hida, Gifu 506-1205 Japan; 3 Department of Physics and Astronomy, University of Bologna and INFN-Bologna, 40126 Bologna, Italy; 4 LPNHE, Sorbonne Université, Université de Paris, CNRS/IN2P3, 75005 Paris, France; 5 Institut für Physik & Exzellenzcluster PRISMA+, Johannes Gutenberg-Universität Mainz, 55099 Mainz, Germany; 6 Institut für Kernphysik, Westfälische Wilhelms-Universität Münster, 48149 Münster, Germany; 7 INAF-Astrophysical Observatory of Torino, Department of Physics, University of Torino and INFN-Torino, 10125 Turin, Italy; 8 Nikhef and the University of Amsterdam, Science Park, 1098XG Amsterdam, The Netherlands; 9 Oskar Klein Centre, Department of Physics, Stockholm University, AlbaNova, 10691 Stockholm, Sweden; 10 Department of Physics and Kavli Institute for Cosmological Physics, University of Chicago, Chicago, IL 60637 USA; 11 Particle and Planetary Physics, New York University Abu Dhabi-Center for Astro, Abu Dhabi, United Arab Emirates; 12 Physik-Institut, University of Zürich, 8057 Zurich, Switzerland; 13 Department of Physics and Astronomy, Purdue University, West Lafayette, IN 47907 USA; 14 INFN-Laboratori Nazionali del Gran Sasso and Gran Sasso Science Institute, 67100 L’Aquila, Italy; 15 Physikalisches Institut, Universität Freiburg, 79104 Freiburg, Germany; 16 Max-Planck-Institut für Kernphysik, 69117 Heidelberg, Germany; 17 SUBATECH, IMT Atlantique, CNRS/IN2P3, Université de Nantes, 44307 Nantes, France; 18 Department of Particle Physics and Astrophysics, Weizmann Institute of Science, 7610001 Rehovot, Israel; 19 LIBPhys, Department of Physics, University of Coimbra, 3004-516 Coimbra, Portugal; 20 Department of Physics “Ettore Pancini”, University of Napoli and INFN-Napoli, 80126 Naples, Italy; 21 Institute for Astroparticle Physics, Karlsruhe Institute of Technology, 76021 Karlsruhe, Germany; 22 Department of Physics and Astronomy, Rice University, Houston, TX 77005 USA; 23 Department of Physics and Chemistry, University of L’Aquila, 67100 L’Aquila, Italy; 24 Department of Physics and Center for High Energy Physics, Tsinghua University, Beijing, 100084 China; 25 Kobayashi-Maskawa Institute for the Origin of Particles and the Universe, and Institute for Space-Earth Environmental Research, Nagoya University, Furo-cho, Chikusa-ku, Nagoya, Aichi 464-8602 Japan; 26 Department of Physics, University of California San Diego, La Jolla, CA 92093 USA; 27 Université Paris-Saclay, CNRS/IN2P3, IJCLab, 91405 Orsay, France; 28 Department of Physics, Kobe University, Kobe, Hyogo 657-8501 Japan; 29 Institute for Subatomic Physics, Utrecht University, Utrecht, The Netherlands; 30 Institute for Advanced Research, Nagoya University, Nagoya, Aichi, 464-8601 Japan; 31 Coimbra Polytechnic-ISEC, 3030-199 Coimbra, Portugal; 32 INFN, Sez. di Ferrara and Dip. di Fisica e Scienze della Terra, Università di Ferrara, via G. Saragat 1, Edificio C, 44122 Ferrara, Italy

## Abstract

The selection of low-radioactive construction materials is of the utmost importance for rare-event searches and thus critical to the XENONnT experiment. Results of an extensive radioassay program are reported, in which material samples have been screened with gamma-ray spectroscopy, mass spectrometry, and $^{222}$Rn emanation measurements. Furthermore, the cleanliness procedures applied to remove or mitigate surface contamination of detector materials are described. Screening results, used as inputs for a XENONnT Monte Carlo simulation, predict a reduction of materials background ($\sim$17%) with respect to its predecessor XENON1T. Through radon emanation measurements, the expected $^{222}$Rn activity concentration in XENONnT is determined to be 4.2 ($^{+0.5}_{-0.7}$) $\upmu$Bq/kg, a factor three lower with respect to XENON1T. This radon concentration will be further suppressed by means of the novel radon distillation system.

## Introduction

The XENONnT detector was constructed for the direct detection of weakly interacting massive particles (WIMPs) [1], a widely discussed dark matter candidate. Additionally, due to the low background levels achieved, it will contribute to a wide array of other rare event searches, such as the two-neutrino double electron capture in $^{124}$Xe [2], neutrino-less double-beta decay of $^{136}$Xe [3], solar-axions [4], and coherent elastic scattering of solar neutrinos [5]. The detector, located in the underground Laboratori Nazionali del Gran Sasso (LNGS), operates as a dual-phase time projection chamber (TPC) with a 5.9 tonnes liquid xenon (LXe) target. Incident particles are observed either through scattering off a xenon nucleus or its electron cloud, which are known as nuclear recoils (NR) and electronic recoils (ER), respectively. XENONnT aims to probe spin-independent WIMP-nucleon cross sections down to $1.4\times 10^{-48}\,$cm$^2$ for a 50 GeV/c$^{2}$ WIMP at 90 % confidence level (C.L.) [6].

To reach the low background requirements for XENONnT, special focus is set on the background mitigation. In this context, a careful selection of the detector materials plays a key role in the suppression of trace radioactive contaminants that contribute to the overall background [7–10]. Detrimental sources of background are the primordial isotopes $^{232}$Th, $^{238}$U, $^{235}$U, $^{40}$K and their progeny, as well as $^{60}$Co and $^{137}$Cs. These contaminants may be inherent to the raw material or get introduced during the production of detector components. Gamma-ray spectroscopy and mass spectrometry are employed to determine the intrinsic radioactivity of construction materials and can reach sensitivities down to 10–100 $\upmu$Bq/kg and 1–10 $\upmu$Bq/kg, respectively.

Another important selection criterion for materials is the emanation rate of $^{222}$Rn. Produced in the decays of residual $^{226}$Ra, which is present in nearly all materials, the radioactive noble gas $^{222}$Rn might be released into the LXe target. There, its progeny can induce low-energy ER background events throughout the sensitive volume. The material selection of XENONnT was aimed to achieve a $^{222}$Rn activity concentration of 1 $\upmu$Bq/kg in the LXe target during standard operation [6].

The long-lived radon daughter $^{210}$Pb and its progeny can also plate-out on material surfaces, mostly before the detector assembly, and thus contribute to the overall background. While the beta decays of $^{210}$Pb and $^{210}$Bi contribute to the so-called ‘surface background’, as observed in XENON1T [11], the alpha-decay of $^{210}$Po can enhance the neutron-induced background through ($\alpha$,n) reactions [6, 12]. A dedicated material surface treatment procedure in combination with cleanroom facilities optimized for storage and detector assembly were developed in order to mitigate radioactive surface contamination.

This paper describes the radiopurity measures applied during the construction of the XENONnT experiment. In Sect. 2, a brief detector introduction is given. A more detailed description can be found in [6]. Section 3 summarizes the results obtained in the gamma screening campaign including complementary mass spectrometry measurements. The radon emanation measurements campaign is described in Sect. 4, while Sect. 5 summarizes the cleanliness measures applied during the detector assembly. The results are summarized in Sect. 6, where the expected background rates, estimated based on the output of the described screening efforts, are discussed.

## The XENONnT experiment

**Fig. 1:**
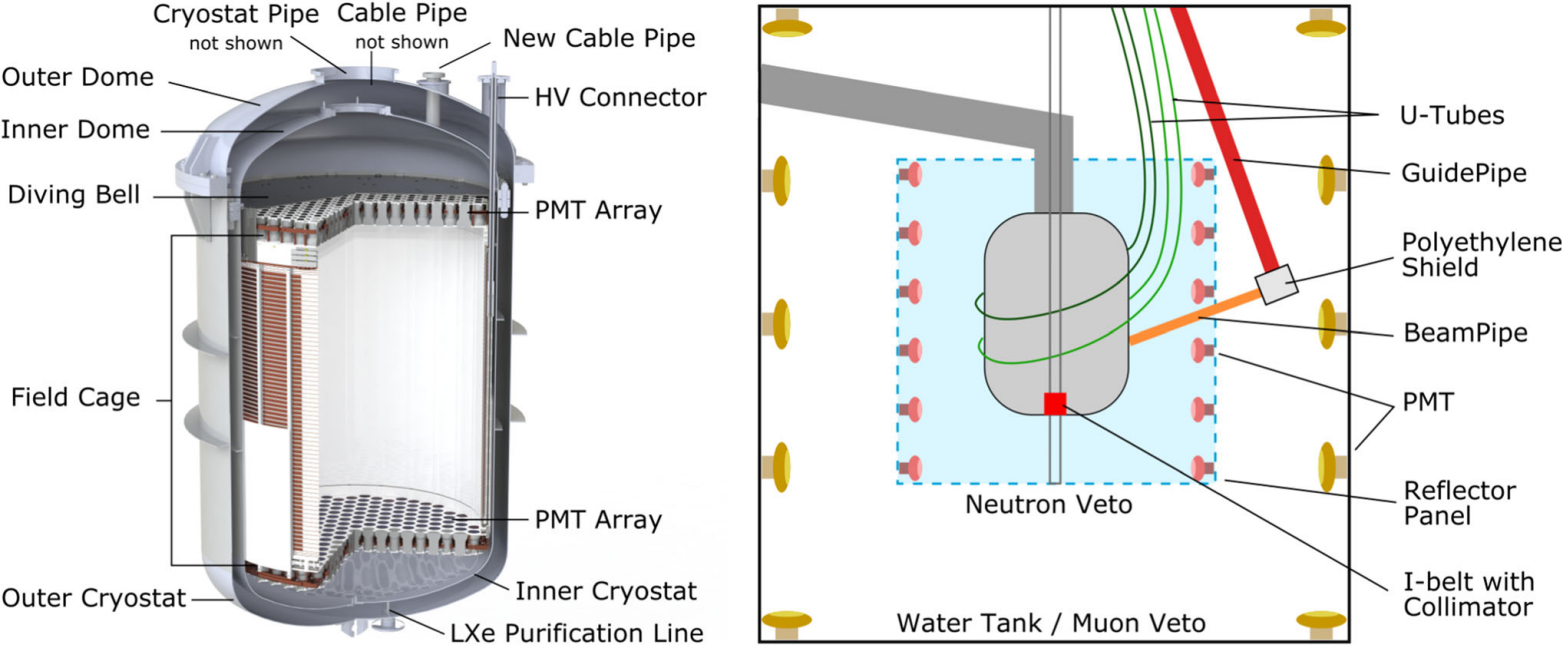
(left) Render of the XENONnT cryostat and TPC, including the most relevant components investigated during the screening campaign. (right) The cryostat is surrounded by the neutron veto and muon veto which serve as shielding for background reduction. The U-Tubes, Guide Pipe, Beam Pipe, and I-belt are part of the calibration subsystem

The XENONnT TPC has a diameter of 1.3 m and a height of 1.5 m. It is housed inside a double-walled vacuum-insulated cryostat vessel, as illustrated in Fig. 1 (left). The TPC is composed of a field cage, electrodes, reflector panels, support pillars, and 494 photomultiplier tubes (PMTs) arranged in two arrays, one each at the top and bottom of the TPC region. Polytetrafluoroethylene (PTFE) reflector panels at the wall provide high reflectivity minimizing the loss of primary scintillation light created in particle interactions in the LXe target. Signal and high voltage (HV) cables of the PMTs are guided to the outside through two cable pipes that are connected to the cable feedthroughs. Vertical PTFE pillars serve as the frame for installing the reflector panels, guard rings, and field-shaping rings. The latter two are made from oxygen-free high conductivity (OFHC) copper and ensure the homogeneity of the electric field needed to drift free electrons toward the liquid-gas interface at the top of the TPC. Once at the interface, a stronger electric field extracts them into the gaseous xenon (GXe) region to create a secondary scintillation signal. The stainless steel (SS) frames of the electrodes (e.g., cathode and anode) hold the parallel electrode wires, with each electrode kept at different potential. The entire TPC hangs from the diving bell, which is immersed in LXe. The LXe level inside the TPC is controlled by pressurizing the diving bell with GXe. The outer insulation cryostat vessel and its flange (outer dome), the functional PMTs, and cables were reused from XENON1T [13].

**Fig. 2:**
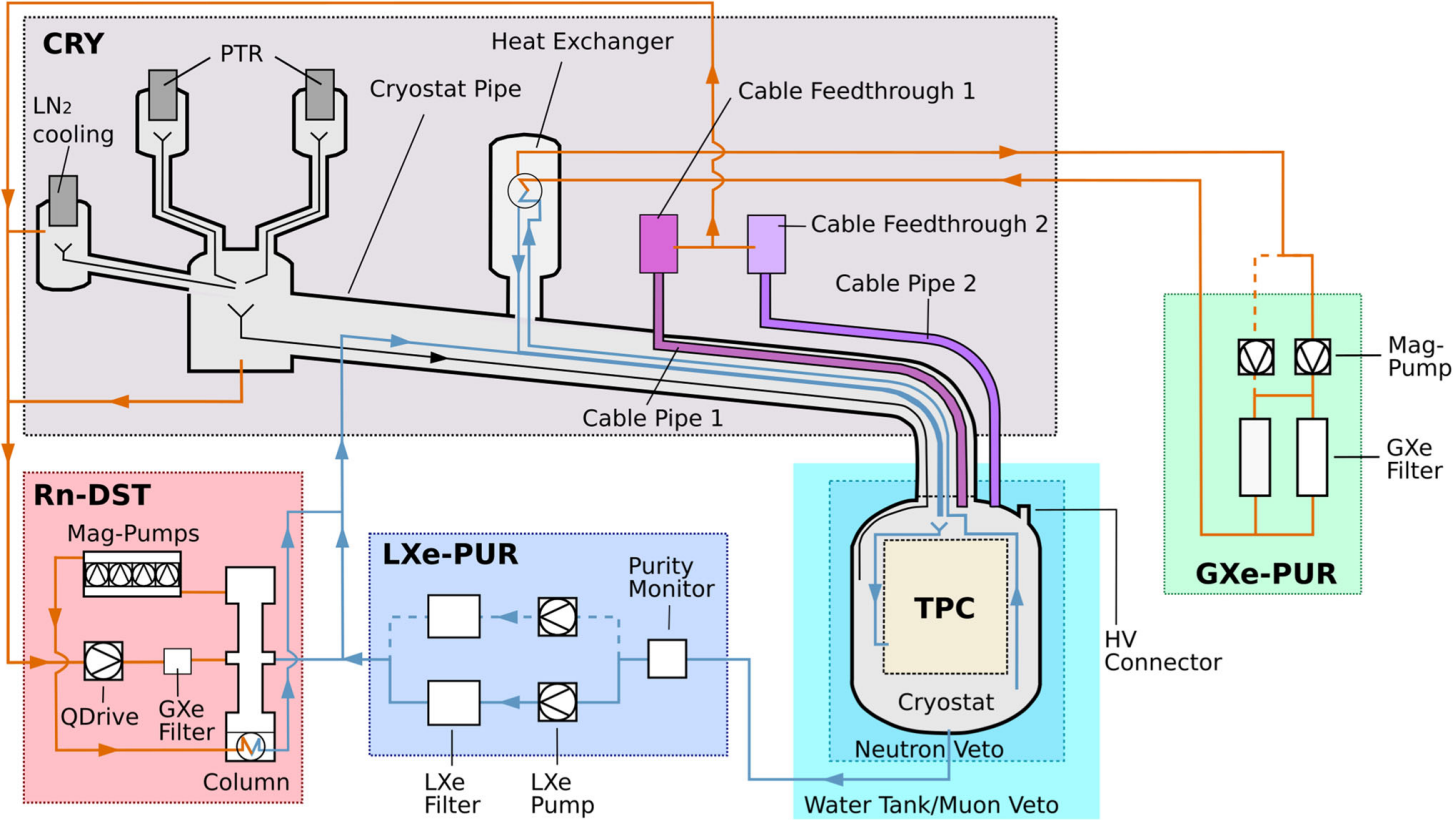
Schematic drawing of the xenon handling system and the TPC of XENONnT. The circulation of xenon through the purification systems is indicated with orange (GXe) and blue (LXe) flow lines

For operating XENONnT, a total xenon inventory of 8.4 tonnes is needed, including 5.9 tonnes of active target mass inside the TPC. The schematics of the xenon-handling system are shown in Fig. 2 (the diving bell has been omitted for better visualization). One major component is the cryogenic system (CRY). It includes two pulse tube refrigerators (PTR) and a liquid nitrogen ($\hbox {LN}_2$) emergency cooling system, which have been retained from XENON1T [13]. The Cryostat Pipe, also entirely reused from XENON1T, connects the cryostat to the rest of the CRY system and houses Cable Pipe 1 and other piping used for xenon purification and liquefaction. The Cable Pipe 2 is placed outside of the Cryostat Pipe as it was installed newly during the upgrade to XENONnT to accommodate additional cabling due to the increased number of PMTs.

Two purification systems constantly clean the LXe target of electronegative impurities. In the GXe purification (GXe-PUR) system, LXe from the cryostat is evaporated and circulated through two high-temperature rare-gas purifiers operated in parallel (GXe Filter in Fig. 2). A magnetically coupled piston pump (Mag-Pump) [14] achieves purification flows of $\sim$50  standard liters per minute (slpm). The same pump is also procuring the GXe to pressurize the diving bell. A second spare Mag-Pump is also available for redundancy.

The new LXe purification (LXe-PUR) system is operated parallel to the GXe-PUR and enables much higher purification flows up to 3 LXe liters per minute (equivalent to 1500 slpm). LXe is extracted from the bottom of the cryostat and flows through vacuum-insulated pipes into the purification unit. A LXe pump (model BNCP-32-000^1^) circulates LXe through a rare-gas purifier material that binds trace amounts of electronegative impurities in the LXe (LXe filter in Fig. 2, using the same reactive material as the GXe filter). Since the LXe filter needs to be regenerated after saturation, the LXe-PUR possesses a redundant LXe pump and filter unit to guarantee continuous operation during the regeneration process. The LXe-PUR is also instrumented with a purity monitor module, which is able to measure the level of electronegative impurities in the LXe coming from the cryostat.

XENONnT is the first experiment to have a dedicated online radon removal system based on cryogenic distillation [15, 16]. While the majority of the purified xenon from the LXe-PUR is returned directly to the cryostat, up to 200 slpm of LXe are directed to the radon distillation column (Rn-DST) to remove trace amounts of radon. Due to the lower vapor pressure of radon compared to xenon, radon accumulates in the LXe at the bottom of the column while the GXe extracted at the top is radon depleted. Four Mag-Pumps [17], similar to the ones used in the GXe-PUR, compress the radon-depleted GXe inside a heat-exchanger which is in thermal contact with the column’s LXe reservoir. There, the radon-depleted xenon is liquefied and returned to the cryostat. In addition to the radon-distillation of LXe coming from the LXe-PUR, there is the possibility to distill GXe extracted from different detector locations. By doing so, radon that is emanated in detector subsystems, such as the cable pipes, gets removed before entering the TPC’s active region. The necessary GXe recirculation flow is achieved with a customized QDrive piston pump from Chart Industries^2^ retained from XENON1T. Electronegative impurities from the GXe are also removed by circulating through a GXe filter unit similar to the ones in GXe-PUR.

For shielding and background mitigation, the cryostat is located inside a water tank, which also functions as an active muon veto and neutron veto (as seen in Fig. 1, right). The water tank and the Cherenkov muon veto system are retained from the XENON1T experiment [13, 18]. The newly-built neutron veto system encloses the region around the cryostat with light reflective panels and is operated with 120 PMTs. Gadolinium-sulfate, which will be dissolved in the entire water volume, efficiently captures neutrons leaving the cryostat [19]. As a consequence of the capturing process, Cherenkov light is produced which is detected.

In addition to the internal calibration sources (e.g., $^{220}$Rn and $^{83\text {m}}$Kr) which are periodically flushed directly into the LXe target, external sources are also used. Two U-tubes extend from the top of the water tank and encircle the cryostat which allow the deployment of external sources close to the detector. Moreover, an I-belt system can be used to move a payload vertically along the cryostat, similar to XENON1T [13]. The I-belt can carry a tungsten collimator containing radioactive sources, e.g. YBe for low-energy neutrons.

Other neutron sources may also be lowered from the top of the water tank inside the Guide Pipe from which collimated neutrons reach the cryostat and TPC through the Beam Pipe. A boronated polyethylene shield surrounds the bottom of the Guide Pipe to reduce neutron captures in the water tank while a neutron source is deployed.

The muon veto, neutron veto, and the calibration systems are not in direct contact with the xenon (see Fig. 1 right) and contribute subdominantly to the overall level of background for dark matter searches. However, since they do contribute to the n-veto background and consequently impact its efficiency, also the materials used for the n-veto and the new calibration system have been screened and carefully selected.

## Radioassay program

The techniques of gamma-ray spectroscopy and mass spectrometry were employed to provide information about the specific activities of radionuclides in detector materials. Acceptable radio-isotopic concentrations for any given batch of raw material vary according to the component’s mass and its proximity to the active region of the TPC. Gamma-ray spectroscopy is a non-invasive method sensitive to a wide range of gamma emitters. Samples with masses ranging from a few grams up to 100 kg were measured with a high-purity germanium crystal for 10–40 days to reach the sensitivity that current subterranean experiments require. Inductively Coupled Plasma Mass Spectrometry (ICP-MS) [20, 21] can determine the composition of a sample by separating and measuring individual isotopes, such as $^{238}$U and $^{232}$Th. The higher sensitivity, as well as the smaller sample sizes and measuring times needed for ICP-MS, make it a complementary method to gamma-ray spectroscopy.

The XENON collaboration employs several highly sensitive germanium spectrometers: Gator [22], GeMSE [23], and four GeMPI spectrometers [24]. The Gator facility and GeMPI detectors sit near XENONnT at LNGS, while GeMSE is located in the Vue-des-Alpes underground laboratory in Switzerland. Each spectrometer is an intrinsically pure p-type germanium crystal (HPGe) set in a coaxial configuration and housed in a low-radioactivity cryostat. The crystal, with a mass of $\sim$2–3 kg, extends into an inner active region enclosed by OFHC copper. The inner chambers are continuously purged with gaseous nitrogen to counteract the influx of ambient radon. The copper is surrounded by 20–25 cm of lead, where the innermost layer has the lowest levels of $^{210}$Pb contamination. These detectors can reach sensitivities of $\sim$10 $\upmu$Bq/kg. GSOr, GeCris and GeDSG, which are part of the SubTErranean Low Level Assay (STELLA) laboratory [25] at LNGS, were also used when needed. They have sensitivities of 1–10 mBq/kg.

Three additional p-type HPGe facilities, Bruno, Corrado, and GIOVE [26] 0.9–1.8 kg, were utilized for smaller components and cleaning agents. These detectors were operated in the underground Low-Level Laboratory at Max Planck Institut für Kernphysik in Heidelberg. These spectrometers are shielded by copper and lead, and are equipped with an active muon veto. In the case of GIOVE, neutron background is further reduced with an additional borated Polyethylene based shielding. These facilities can reach sensitivities of 0.1–1 mBq/kg.

Samples screened in the HPGe facilities were cleaned with mildly acidic soap (e.g. Elma clean 65), rinsed with deionized water (DI water), and immersed in ethanol (>95%). Both steps were completed with a 20-minute ultrasonic bath (US-bath). If a sample could not be cleaned with acidic soap or immersed in liquid (e.g. photomultipliers and cables), its surface was wiped thoroughly with ethanol. All samples were stored within clean plastic bags to mitigate plate-out of radon daughters during transport. Prior to the measurement it was verified that all traces of ambient radon and radon daughters have been removed or decayed by monitoring the count rates of the associated gamma lines.

The Geant4 toolkit [27] was used to simulate each individual sample inside the respective HPGe spectrometer in order to ascertain the detection efficiency for each gamma line. The specific activities (or upper limits) were then calculated from data based on the sample’s mass and measuring time, as well as the characteristic branching ratios of the gamma lines, as detailed in [22].

The complementary analytic technique ICP-MS is among the most sensitive for the detection of trace elements. The intrinsic radioactivity of a batch of material can be found with a measurement of long-lived radionuclides. The sample is turned into an aqueous solution, introduced through a peristaltic pump, nebulized in a spray chamber, and then atomized and ionized in plasma. These ions are extracted into a system placed under high vacuum and separated in accordance with the charge-to-mass ratio. Sensitivities 1–10 $\upmu$Bq/kg are attainable for $^{238}$U and $^{232}$Th.

Several live years of data, aggregated across all instruments, were acquired throughout the radioassay program of XENONnT. Relevant measurements for detector construction are covered here, where more supplemental data is available in [28]. For detected activity, the 1$\sigma$ uncertainties are given, including both statistical and systematic contributions. Systematic uncertainties result primarily from efficiency simulations. Otherwise, upper limits are provided at 95 % C.L. In the case of ICP-MS, uncertainties are given to account for instrumental precision, calibration, and the recovery efficiency. A break in secular equilibrium in the $^{238}$U decay chain is identified by comparing the results obtained for $^{238}$U and $^{226}$Ra from gamma spectroscopy. A deviation from the $^{228}$Ra result from the direct measurement of $^{232}$Th with ICP-MS indicates a break in secular equilibrium in the $^{232}$Th chain. The results of materials and cleaning agents (discussed in Sect. 5.1) selected for use in the XENONnT TPC and cryostat are shown in Table 2 in Appendix section. Components fabricated from these materials are displayed in Fig. 1 (left). These components contribute substantially to the overall background rate of XENONnT. These results are incorporated into the sensitivity study of XENONnT [6] through a Monte Carlo simulation of the material-induced background. Whenever only an upper limit is available, the upper limit is assumed as the activity to acquire the most conservative sensitivity.

Similarly, Table 3 in Appendix section lists the specific activities of components selected for usage in the neutron veto, calibration and purification subsystems. These components do not contribute significantly to the materials-induced background for the dark matter search, but they determine the acceptable tagging window and coincidence threshold for the PMTs of the neutron veto system.

Overall, the inherent concentrations of isotopic impurities in the bulk materials used in XENONnT are comparable to the materials used in XENON1T [9]. Some of the PTFE (Sample 9) from which TPC wall reflectors were fabricated proved to be higher than expected in $^{40}$K but lower in $^{238}$U, $^{226}$Ra and $^{137}$Cs. The remaining wall reflectors (Sample 10), on the other hand, were made of material low in $^{40}$K and $^{137}$Cs, but higher in $^{226}$Ra. The SS material selected in XENONnT for the electrode frames, bell, and inner cryostat vessel, came after several batches were rejected because they would have contributed substantially to the NR background through spontaneous fission and ($\alpha$,n) reactions. The selected SS material also exhibited low specific activities of $^{60}$Co ($\sim$1 mBq/kg), which is the most substantial contributor to the ER background in that material. Much of the dedicated efforts were additionally focused on the individual components of the PMTs. Most of the components showed similar levels of impurities to those in XENON1T [29]. Results for the ceramic stems are given in Table 2 in Appendix section as they are the largest contributor to the total radioactivity of the PMTs. OFHC copper did not exhibit any deviation from the expected purity. Multiple earlier samples of Gadolinium Sulfate proved to have higher concentrations of all isotopes, except $^{137}$Cs, by 1–2 orders of magnitude. Table 4 in Appendix section lists the results for example materials that were rejected in the course of the radioassay program. A comparison of spectroscopic and spectrometric results shows no significant break in secular equilibrium in the $^{232}$Th chain for any screened material.

## $^{222}$Rn emanation measurements

The emanation rate of $^{222}$Rn from detector components cannot be inferred from gamma-screening due to the often unknown radon diffusion in materials and the potential inhomogeneous distribution of the mother isotope $^{226}$Ra. Thus, prior to the construction of XENONnT, all detector materials were investigated for their radon emanation. Furthermore, fully assembled detector subsystems were measured for radon emanation in order to get a complete understanding of the locations of radon sources in the system. This information is needed to optimize the performance of the radon distillation system.

The results in this section refer to the $^{222}$Rn activity at its emanation equilibrium and are given with a combined uncertainty including statistical and systematic errors, unless specified otherwise. If a result is compatible with zero within 1.645 $\sigma$, a 90 % C.L. upper limit is given instead.^3^

### $^{222}$Rn assay technique

The applied $^{222}$Rn assay techniques are described in detail in [10]. The investigated sample was left for several days inside a gas-tight vessel filled with a radon-free carrier gas at ambient temperature. Emanated $^{222}$Rn atoms from the sample accumulated in the carrier gas during this emanation time. Then, the carrier gas was pumped through a $\hbox {LN}_{2}$-cooled adsorbent trap, the so-called radon trap, where the radon was collected and separated from the carrier gas. In case of an equilibrium between radon emanation and its decay, the activity of the trapped radon corresponds to the sample’s emanation rate. It was measured using miniaturized proportional counters which reach sensitivities down to $\sim \,$20 $\upmu$Bq [10, 30]. If the sample had been exposed to xenon prior to the measurement (e.g., all reused XENON1T systems), large xenon outgassing rates prevented the usage of the proportional counters, as the xenon gets collected in the radon trap as well. As a consequence, the proportional counter’s active volume of $\sim \,$1 cm$^{3}$ was too small to house the entire sample.

In that case, electrostatic radon monitors were used which have significantly larger volumes ($\sim \,10^{3}$ cm$^{3}$) and sensitivities of $\sim \,$0.1 mBq [31, 32]. The radon monitor does not detect the $^{222}$Rn decay directly, but its alpha-decaying daughters $^{218}$Po and $^{214}$Po which, due to an electric drift field, are collected on a $\alpha$-sensitive photodiode. The detection efficiency for each daughter isotope can be different, but they both strongly depend on the gas composition. Outgassing impurities released from samples have been shown to impact the detection efficiency of radon monitors [33]. For this reason, after each measurement a calibrated amount of radon was added to the sample for a calibration in the present gas composition.

Xenon outgassing may also hinder the extraction of the carrier gas as it freezes inside the radon trap and blocks the gas flow. Such an effect was already observed during the XENON1T radon screening campaign [10]. Therefore, the $^{222}$Rn collection was done in a 2-stage approach: Before the radon trap another trap was included, the so-called xenon trap. It is a SS vessel filled with copper wool and held at LN2 temperature during the extraction. Xenon and the majority of radon freezes out in this trap however, due to the loose packing of the copper wool and the relatively large cross section of the trap, the carrier gas flow hardly degrades. All radon (and also xenon), which cannot be stopped in the xenon trap is collected in the subsequent radon trap which is also held at LN2 temperature. At the end of the extraction, the xenon trap is warmed and its entire content is transferred into the radon trap. With this extra step, the entire procedure doesn’t suffer from any flow degradation due to a blocked trap and all extracted radon from the sample can be stored in the radon trap (as verified using a calibrated radon source). In case of large gas samples to be extracted, the pumping power through the radon trap was too weak to extract the entire sample. Then, the quoted activities are corrected for this reduced extraction efficiency assuming that the emanated radon was homogeneously distributed within the carrier gas prior to extraction. This correction is referred to as scaling. In order to generate a homogeneous radon distribution in large volume samples, the carrier gas was mixed prior to the sample extraction by adding additional clean carrier gas via multiple filling ports.

### Radon emanation of construction materials

All the results of this section can be found in Table 5 in Appendix section. Almost 14 km of PMT signal read-out and HV cables run from the TPC to the cable feedthrough, through the CRY system. Several samples of HV cable from the company Accu-Glass were measured, all of them with emanation rates well-within the requirements (Rn1, Rn2  and Rn3). For the signal cables, initially a PTFE-insulated coaxial cable from HABIA was considered (item Rn4, same company and type as item #42 in [10]), but it was found to emanate a factor $\sim \,$50 more than the sample reported in [10]. Alternatives from two different companies were explored instead: three signal cable samples from the company Huber+Suhner (Rn5, Rn6  and Rn7) and one from the company Pasternack (Rn8). All of them gave similar results, well-within the requirements. The cable’s emanation results given above serve as an upper limit for the expected emanation of all cables (HV and signal cables) enclosed inside Cable Pipe 2 and the cryostat (the emanation of cables inside Cable Pipe 1 was already studied in [10]). A measurement of their final activities will be presented later.

The inner pistons of all Mag-Pumps are sealed from their outer cylinders by a plastic-type gasket. An ultra high molecular weight polyethylene (ULHWP, used for the Mag-Pump in XENON1T [14]) was measured (Rn9). Several alternative gasket materials were explored (Rn10, Rn11 and Rn12). All considered options showed negligible contributions with respect to the expected overall radon emanation of the pump (based on sample #23 in [10]).

The radon emanation from individual components from the two LXe pumps was measured separately. Results are shown as Rn13-Rn16 . The SS cryogenic valve and aluminum rotor were found to contribute negligibly while Viton O-rings might contribute notably to the total emanation of a LXe pump. Here it should be noticed that only a fraction of the O-ring’s surface is expected to emanate radon into the LXe volume.

For the oxygen removal in the LXe-PUR system, a filter material made from copper electrolytically deposited onto alumina balls was considered. Different batch samples of the same filter material were investigated for their radon emanation (samples Rn17 and Rn18). They also appear in Table 2, samples 48-50, where the differences observed in $^{222}$Rn emanation rates can be related to the $^{226}$Ra activities. While this filter has a very high oxygen removal rate, its radon emanation is at least a factor of 100 larger than the eventually used LXe filter discussed below.

### XENONnT radon emanation activity

The radon emanation rate of all detector subsystems introduced in Fig. 2 was measured separately. The results are listed in Table 6 in Appendix section. Based on these measurements, the locations of all relevant radon sources were identified. Some of the samples, or even entire subsystems of XENONnT, were already measured in preparation of the XENON1T experiment. Their emanation results reported in [10] are also included here.

An important result is the emanation from the SS inner cryostat (Rn19), where only surfaces facing inwards contributed to the measurement. Despite having a five times larger surface area, the emanation rate is comparable with the one obtained for the XENON1T inner cryostat (sample #48 in [10]). The XENON1T cryogenic system was reused for XENONnT, and was kept under a nitrogen atmosphere during the detector upgrade. The result of an integral measurement (Rn20) of the Cable Feedthrough 1, Cable Pipe 1 and the reused cooling towers of XENON1T was consistent with the emanation rate measured in [10] (obtained by adding together samples #13, #14 and #49-#52). The Cable Feedthrough 2 and the Cable Pipe 2 were measured for the first time (Rn21 and Rn22, respectively). Compared to Cable Pipe 1 (#49 in [10]), Cable Pipe 2 has a factor $\sim \,$3 lower emanation rate. This is consistent with the fact that total length of cable enclosed in Cable Pipe 2 is smaller with respect to Cable Pipe 1. The integral rate of CRY is 21 (3) mBq, excluding the emanation from the XENONnT TPC, which is discussed later.

The two GXe filter units of the GXe-PUR system were measured in their hot operating state (#16  and #17  in [10]). The emanation from the Mag-Pump 1 was taken from #23  in [10] as it was used already in the final science run phase of XENON1T. The integral emanation value of the GXe-PUR system of 1.7 (2) mBq, was obtained by adding the radon emanation from all individual parts. The emanation of the second redundant Mag-Pump, serving as a backup system, is not considered.

**Fig. 3:**
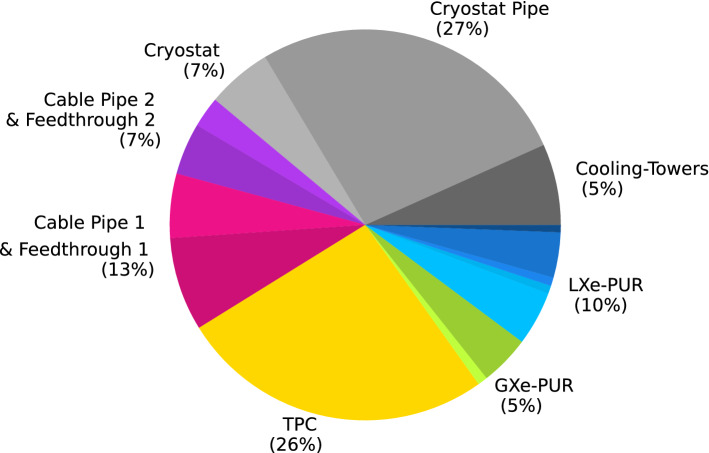
The different subsystem contributions to the overall $^{222}$Rn emanation rate in XENONnT, adding up to 35.7 ($^{+4.5}_{-5.9}$) mBq. The colors correspond to the scheme used in Fig. 2. Only central values from Table 6 in this work and Figure 2 in [10] have been used. The radon emanation from the Rn-DST system is not taken into account

The LXe-PUR was split in six volumes which were measured separately. One volume (Rn23) included the majority of vacuum insulated piping for the LXe and cryogenic valves. The second volume (Rn24) contained the purity monitor which showed a similar emanation rate as the LXe filter 1 in the third volume (Rn25). A 500W Xe-$\hbox {N}_{2}$ heat-exchanger was the main component of a forth volume (Rn26) which was measured to have a five times higher emanation rate than the previous three. The two last volumes contained one LXe pump each (Rn27). Both pumps gave similar results and were found to contribute to $\sim$44% of the total radon emanation of LXe-PUR. Further investigations identified the pump’s main-body made from SS as the main contributor. Both pumps were dismounted and the main body of the pump was electropolished. Earlier investigations showed a factor greater than three improvement may be achieved by electropolishing [10]. A measurement of the pump’s emanation rate after this treatment is not available. Therefore, the integrated result of 3.6 (2) mBq for the LXe-PUR does not reflect the potential reduction and thus can only be seen as an upper limit.

An integral measurement of the Rn-DST system after assembly was not possible and the emanation rate of some single components remains undetermined. The emanation of those items is estimated based on previous representative samples such as items #4-9 in [10] for SS and results from Table 1 in [30] for copper, 150 (100) $\upmu$Bq/m$^{2}$ and 1.2 (0.2) $\upmu$Bq/m$^{2}$, respectively. For the estimation of the distillation column’s radon emanation rate (Rn28), we consider the emanation from the eight SS packing-material pieces which fill the interior of the column. This packing material is the main contributor to the internal surface of the column. Their emanation rate is derived from the measurement of three packing-material pieces, given in Table 1 (Pack 4/5/6 combined). The contribution of other SS and copper surfaces in the distillation column to the total emanation rate is considered to be negligible. Emanation from QDrive pump and GXe filter unit were also taken into account (#20$_{a}$ in [10], and Rn29). It should be noted that emanated radon atoms from the column, QDrive and the GXe filter 3 are expected to never reach the LXe target due to the distillation process. Radon sources located downstream of the column, and thus after the radon removal process, are the four Mag-Pumps (Rn30), the related tubing (Rn31, Rn32  and Rn33) and a heat exchanger (Rn34) where radon-depleted xenon gas is liquified. The integral value of Rn-DST system is 1.6 (2) mBq.

The most challenging measurement for XENONnT was the fully assembled TPC. In the absence of a dedicated gas-tight vessel, the TPC could only be measured once enclosed in the cryostat together with the rest of the CRY system. Hence, the TPC emanation rate is an indirect measurement with respect to sample *Integral CRY* in Table 6. The measurement procedure was the same as for the rest of the samples described in this work (see Sect. 4.1); however, due to the large PTFE surfaces and the expected outgassing, some extra precautions were taken in order to minimize the impact on the radon monitor’s detector efficiency. Firstly, before the measurement started, the entire CRY system and TPC were kept under vacuum pumping for several weeks to reduce the outgassing rate. Secondly, a commercial $\hbox {N}_{2}$ gas purifier was installed at the extraction port such that the extracted $\hbox {N}_{2}$ carrier gas was further cleaned of impurities before reaching the radon trap. The averaged TPC emanation rate obtained from three separate, consecutive radon extractions is 9.3 (3.8) mBq.

For the TPC measurement, the carrier gas was extracted through several ports located at the bell, Cable Feedthrough 2, Cryostat Pipe and cooling towers. The amount of gas extracted via each individual port was varied between the three measurements. An active mixing of the carrier gas prior to extraction was not possible. Thus a non-homogeneous radon concentration potentially influenced the TPC result due to the applied scaling. This systematic uncertainty was estimated in a numeric simulation that accounts for the known emanation rates of detector subsystems and other details about the extraction procedure (i.e., the locations of the extraction ports and their individual gas flows). The simulation was developed to probe different scenarios of the dynamics of the carrier gas before and during the extraction, ranging from a laminar flow to a turbulent mixing of the carrier gas in all detector subvolumes. The final TPC emanation rate, based on this systematic uncertainty study, was found to be 9.3 (3.8)*stat* ($^{+1.2}_{-4.6}$) *sys* mBq (Rn35 in Table 6 in Appendix section). To put this value into context, the emanation from the 494 PMTs is expected to be of $\sim \,$2.2 mBq (from #37 and #38 in [10]), and the emanation from cables running inside the inner cryostat add additional $\sim \,$3 mBq (as discussed already in Sect. 4.2). The emanation from other TPC materials, such as PTFE or copper, was not measured prior to assembly.

Taking into account the presented radon emanation measurements for the different xenon handling systems, the total $^{222}$Rn  emanation  rate  of  XENONnT is estimated to be 35.7 ($^{+4.5}_{-5.9}$) mBq. It should be noted that this number is estimated from measurements performed at ambient temperature where the radon emanation rate might be increased with respect to the emanation at the detector’s operating temperature. For XENON1T, however, this effect was not observed as the measured radon concentration in the operating TPC was about 30% higher with respect to the expectation from radon emanation measurement [10]. A further $^{222}$Rn reduction due to the operation of the Rn-DST system is not considered here. Figure 3 shows how the sources contribute to the overall radon budget. Assuming a homogeneous distribution of radon in the entire xenon inventory, the total radon emanation rate translates to an activity concentration of 4.2 ($^{+0.5}_{-0.7}$) $\upmu$Bq/kg. The radon concentration in LXe is expected to be reduced by the operation of Rn-DST, bringing the XENONnT target value of 1 $\upmu$Bq/kg within reach.

## Surface treatment

A thorough cleaning of materials serves multiple purposes. It removes small particulates that, if released inside the detector, might compromise the detector’s operation, in particular the HV stability of the electrodes. Furthermore, the cleaning process removes grease and lubricants that may be left over from the manufacturing process. An adequate chemical treatment also leads to controlled passivation and surface conditioning of delicate metallic surfaces, such as the detector’s fine electrode wires [34]. Finally, a dedicated cleaning procedure can help to remove radioactive isotopes that have been accumulated on material surfaces during production, storage and handling. This section summarizes the cleanliness efforts in preparation for the XENONnT detector, including cleaning procedures, infrastructure, and the measures taken to avoid re-contamination during detector assembly.

### Cleanroom infrastructure

For material cleaning, storage and detector assembly, two cleanrooms (CRs) at the LNGS laboratory were utilized. The above-ground cleanroom (AG-CR) was located inside an assembly hall and had a footprint of $(9\times 5)$ m$^2$. The ambient air was cleaned using HEPA filters and then flushed with laminar flow through the CR. Periodic particle counter measurements demonstrated ISO 6 classification. All of the large-scale detector materials, including the TPC, were cleaned in the AG-CR. In order to maintain cleanliness, all materials entered the cleanroom via an anteroom where pre-cleaning happened (see Sect. 5.2). Large-scale items were brought directly into the AG-CR through a gate bypassing the anteroom. Thanks to the adjusted air flow and movable curtains, which were mounted before opening the gate, a temporary anteroom could be established for the pre-cleaning of these large items.

**Fig. 4:**
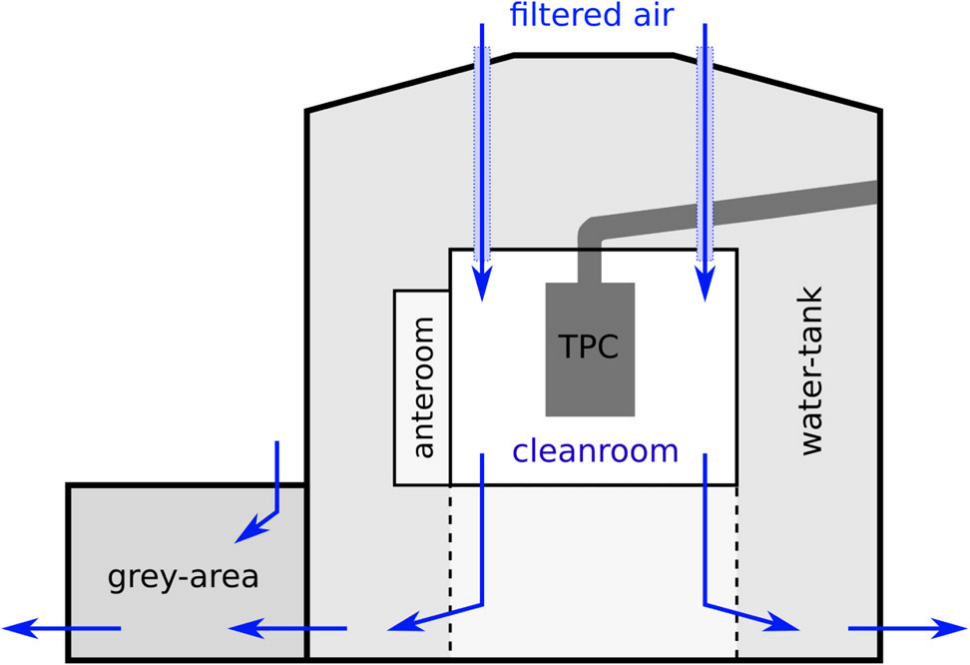
The UG-CR (ISO 6 class) is built around the cryostat in the center of the water tank. Filtered air is flushed first into the cleanroom in the center and is pushed thereafter through the water tank into the grey area, providing a sequence of decreasing cleanliness levels toward ambient air

After assembly in the AG-CR, the TPC was protected against contamination (see Sect. 5.3) and transported to the experimental site underground for installation. In order to guarantee a clean installation, a dedicated underground cleanroom infrastructure (UG-CR) of different cleanliness levels was built inside XENONnT’s water tank, as shown in Fig. 4. The UG-CR was accessible through a so-called grey area that was temporarily built in front of the water tank during the construction phase of XENONnT. Constantly flushed with filtered air, it was used to clean materials and equipment before entering the CR. From the grey area, one could access the water tank, which as a whole was provided with filtered air at a slightly higher pressure than in the grey area. The water tank was treated as a clean environment. Materials and equipment foreseen for the CR were cleaned or unpacked here.

The actual CR had a footprint of $(5\times 5)$ m$^2$ and was located at the center of the water tank. It enclosed the XENONnT cryostat after the cryostat’s assembly. Filtered air was flushed directly into the CR, guaranteeing a certified ISO 6 classification. From there, the clean air was guided through the water tank and the grey area following the decreasing cleanliness levels of those areas. When needed, the area right below the CR could be separated from the rest of the water tank by movable plastic curtains (shown with dashed lines in Fig. 4). The floor of the CR was then opened to lift the assembled TPC or the inner cryostat vessel up into the CR. The filtered air was flushed with high flow rate from the CR through the separated area underneath to maintain the air quality throughout this temporarily enlarged volume.

### Cleaning procedures

All final cleaning happened in a clean environment, mostly in the AG-CR. Deionized water with an average electrical conductivity of $\sim \,$0.08 $\upmu$S/cm was supplied by a dedicated water plant with a flow up to $1.3\,$m$^3$/h. For the cleaning of large-scale items, the AG-CR was equipped with two containers made of high-density polyethylene (HDPE) with a $(1.9\,\times 1.6\,\times 0.4)\,$m$^3$ volume and a slightly larger container of the same type. The latter was used as a custom-made US-bath and housed four US transducers each with a $(0.5 \times 0.7)\,$m$^2$ surface area and a maximum power of $2\,$kW at a frequency of 40 kHz. Two heaters of $20\,$kW in total were available to bring the baths to the required temperatures. A custom-made crane was built to handle heavy items during cleaning. Prior to bringing detector materials, tools, and containers into any CR, they were pre-cleaned with ethanol-soaked wipes or neutral soap. Further decreasing followed as a first cleaning step inside the CR. Therefore, detergents were selected depending on the materials to clean but also with respect to their internal radioactivity (see Table 2 in Appendix section). Alkaline detergents such as HARO Clean 188^4^ (item 34 in Table 2 in Appendix section) showed high $^{40}$K activities of up to several kBq/kg and were not applied to soft or porous materials such as PTFE, which might absorb small amount of the solution including the radioactive impurities. For stainless steel, gamma spectroscopy measurements did not indicate an increase of the material’s activity after the usage of such solutions. Attention was also paid to the detergents’ $^{226}$Ra contamination. Being the progenitor of $^{222}$Rn, radium plating out on the material surfaces during the cleaning process could significantly increase the radon emanation rate. For a sample of P3-Almeco 36,^5^ a $^{226}$Ra contamination of $98(27)\,$mBq/kg was found (see item 36 in Table 2 in Appendix section), the highest of all screened detergents. Indications for the plate-out of radium were observed during the cleaning process of samples of the SS packing material used in the cryogenic radon distillation column (see Table 1).

**Table 1:** Radon emanation results of distillation packing material before and after selected cleaning procedures

Sample	Procedure	Emanation rate (mBq/piece)
Pack 1	No cleaning	0.13(4)
	Almeco treatment	0.80(5)
Pack 2	Almeco treatment	1.30(6)
	additional DI water rinsing	0.92(7)
	repeat additional DI water rinsing	0.86(10)
Pack 3	Almeco treatment	2.58(15)
	repeat Almeco treatment	3.26(18)
Pack 4/5/6 combined	Acetone treatment	0.21(3)

After a 10-minute bath in 5% P3-Almeco 36 solution at $60\,^\circ$C, one sample’s radon emanation rate increased by a factor of six to $0.80(5)\,$mBq (Pack 1 in Table 1). Other samples (Pack 2 and Pack 3) also showed an emanation rate significantly higher than that of the untreated samples. In the case of Pack 3, the repetition of the P3-Almeco 36 cleaning procedure further increased its emanation rate. Attempts to use DI water to flush out or dilute potential detergent residuals from the largely inaccessible, heavily folded surface yielded only a small reduction (Pack 2). The packing material was cleaned in acetone instead, for which no $^{226}$Ra contamination was detected after treatment. For the cleaning of other large volume materials, mostly Elma clean 65^6^ was used (see item 38 in Table 2 in Appendix section), the detergent with the lowest measured $^{226}$Ra contamination, as well as a mixed solution from the detergents HARO Clean 188 and HARO Clean 106 (see items 34 and 35 in Table  2 in Appendix section).

After the first detergent treatment, most detector parts were cleaned in an acidic solution adjusted to their material composition, as shown below. Depending on the material, the purpose of this cleaning step is either to remove a thin surface layer from the material, and thus its contamination, or to dissolve impurities in the solution. The purity level of the chemicals was >99% (pro analysis). A gamma spectroscopy measurement of a nitric acid sample was used to set upper limits on its radiopurity, most importantly for $^{226}$Ra (see item 40 in Table 2 in Appendix section). The detailed procedures for different types of materials are specified below.

#### Copper

Copper cleaning was developed based on a procedure described in [35] for the removal of surface contamination while retaining surface details such as threads or boreholes.

- Elma clean 65 neutral soap (5%), $15\,$min at 35  –  40 $^\circ$C in US-bath
- thorough DI water rinsing
- $\hbox {H}_2$ $\hbox {SO}_4$ (1%) + $\hbox {H}_2$ $\hbox {O}_2$ (3%) solution, $5\,$min at room temperature
- immerse in DI water bath
- citric acid (5%), $5\,$min at room temperature
- thorough DI water rinsing
- cleanroom wipes and $\hbox {N}_2$ blowing for drying

A re-deposition of dissolved copper in the sulfuric acid solution could be prevented by moving the copper pieces throughout the washing. The drying process needed to happen immediately after the final rinsing to prevent a rapid oxidation of the copper. For large items, an increased temperature of the last rinsing bath ($\sim 35^{\circ }$C) supported this drying process with its implied higher evaporation rate.

#### PTFE and other plastics

PTFE is known to attract positively charged radon daughters and thus promote their plate-out on its surface [36]. In order to increase the reflectivity of vacuum ultraviolet light, the PTFE surfaces that face the inner LXe volume have been shaved with a diamond-tipped tool. During this process up to 1.5 mm of surface layer was removed. Thus, impurities located on the material surface or just below were removed, making the shaving process an important cleaning step. This effect was studied using the XIA UltraLo spectrometer,^7^ located in the underground laboratory at Kamioka [37] which measured the surface activity of $^{210}$Po on PTFE samples. For an unshaved reflector sample, an activity of $126(8)\,$mBq/m$^2$ was measured, while a reduction to 20(3) mBq/m$^2$ was observed on the PTFE-surface treated with the diamond-tipped tool. Only the surfaces of PTFE reflector panels facing the TPC underwent the shaving process.

All PTFE parts, but also other plastics such as Kapton and PEEK were chemically cleaned according to the procedure outlined below. The capability of different cleaning procedures to remove radon daughters from PTFE surfaces was studied in [38]. It was shown that the contamination of $^{210}$Po and $^{210}$Pb could be reduced by a factor two at most, almost independent of the chemicals used. This indicates that only radon daughters that have not been implanted into the bulk PTFE can be removed. A better reduction factor of $\sim \,$30 has been achieved for the removal of $^{212}$Pb from PTFE surfaces [38]. Thus, the following nitric acid based procedure was used:

- Elma clean 65 neutral soap (5%), $15\,$min at $35-40\,^\circ$C in US-bath (no US used for diamond-shaved parts)
- thorough DI water rinsing
- $\hbox {HNO}_3$ (5%) solution, $2\,$h at room temperature including 15 min in US-bath (no US used for diamond-shaved parts)
- immerse in DI water bath up to 1 h to dissolve acid residuals
- thorough DI water rinsing
- $\hbox {N}_2$ blowing for drying

For the diamond-shaved surfaces of the PTFE reflector panels the US-bath was not used to avoid the risk of damaging the treated surfaces. A dedicated storage in nitrogen-flushed boxes helped to mitigate the re-contamination of the PTFE with radon daughters (see Sect. 5.3) and removed residual humidity.

#### Electrodes and other stainless steel items

The five electrodes of XENONnT are made from SS wires of diameters 216 $\upmu$m and 304 $\upmu$m, fixed to a corresponding SS electrode frame. Before assembly of the electrodes, the wires were mounted on a holding structure designed for the cleaning process. The electrode frames themselves were cleaned separately, using the same procedure as for the wires. As a detergent, a mixture of the alkaline HARO Clean 188 and the cleaning amplifier HARO Clean 106 was used. A positive effect of nitric acid (35%) on the emission of single electrons from electrodes was documented in [34]. For safety reasons, however, 7% citric acid solution was used instead, an alternative approach that is common in industry for SS passivation when nitric acid cannot be utilized. The detailed procedure is:

- HARO Clean 188 (5%) + HARO Clean 106 (0.01%) solution, $10\,$min at 45–50 $^\circ$C in US-bath
- thorough DI water rinsing
- Citric acid (7%) solution, $1\,$h at $45-50\,^\circ$C including 10 min in US-bath
- thorough DI water rinsing
- storage in $\hbox {N}_2$ flushed boxes for drying

After assembly, the wired electrodes were cleaned again in Elma clean 65 (5%) solution for 15 min at room temperature, without US. Other small SS parts were treated only with the HARO Clean 188/106 detergent mixture (first step in the procedure above). Large SS items were electropolished by the manufacturers beforehand in order to remove surface contamination [39, 40]. The inner cryostat vessel was cleaned with ethanol-soaked wipes after its electropolishing.

#### PMTs and cables

During the decommissioning of the XENON1T detector, its PMTs were dismounted and sealed in a CR environment for later use in XENONnT. All PMTs, including the newly purchased ones, were cleaned inside the AG-CR before the detector assembly. The procedure included:

- $\hbox {N}_2$ blowing to remove small particulates
- soft wiping of insensitive parts using ethanol
- immersion of the PMT in an ethanol bath

HV and signal cables have been soldered to the PMT bases beforehand. Therefore, cabels and bases needed to be cleaned together. For better handling, the bases were mounted on acrylic structures to keep them in place throughout the cleaning process which included the following steps:

- thorough DI water rinsing to remove dust particles before entering the AG-CR
- immerse only the cables in Elma clean 65 soap (5%), 15 min at 30$\,^{\circ }$C in US-bath, the bases stayed outside the bath
- immerse also bases in Elma clean 65 soap (5%), 15 min at 30$\,^{\circ }$C without US
- thorough DI water rinsing
- $\hbox {N}_2$ blowing for drying
- immerse cables and bases in ethanol bath to accelerate the drying process

After drying, cables and bases were wrapped and stored inside the CR until assembly.

Once installed in the TPC, the cables, which are directly connected to the PMT bases, reach only from the PMT array to the opening of the two cable pipes at the inner dome of the cryostat (see Fig. 2). There they were connected to cables which had been pre-installed inside the two cable pipes. The cables in the cable pipe 1 were reused from XENON1T and kept under nitrogen atmosphere during the detector upgrade so that no further cleaning was necessary. The cables in the new Cable Pipe 2 were thoroughly wiped with ethanol inside a CR before being installed in the pipe.

### Plate-out of radioactive impurities and material storage

Detector materials must be protected and stored properly to maintain cleanliness. Besides the plate-out from generic dust a particular emphasis was placed on mitigating contamination from radium and radon daughters on PTFE surfaces. In order to estimate the plate-out rate of long-lived radon daughters, PTFE plates of 20 cm$^2$ surface area were placed at various locations in the CRs and at the XENONnT experimental site. By means of alpha spectroscopy [38] an upper limit for the increase of the surface activity of $^{210}$Po due to plate-out of <19 mBq/d/m$^2$ (95% C.L.) was determined. To investigate potential radium plate-out e.g. from dust in ambient air, two PTFE samples, each of $3\,$m$^2$ surface area, were exposed to ambient air at two different locations . After 105 days of exposure, the increase of the $^{222}$Rn emanation rate due to the radium plate-out was measured by employing the radon assay technique described in Sect. 4. For the sample placed at the XENONnT experimental site, an increase of the $^{222}$Rn emanation rate of 0.28(15) $\upmu$Bq/d/m$^2$ was observed. The averaged radon concentration in the ambient air during the exposure time was $36\,$Bq/m$^3$.

**Fig. 5:**
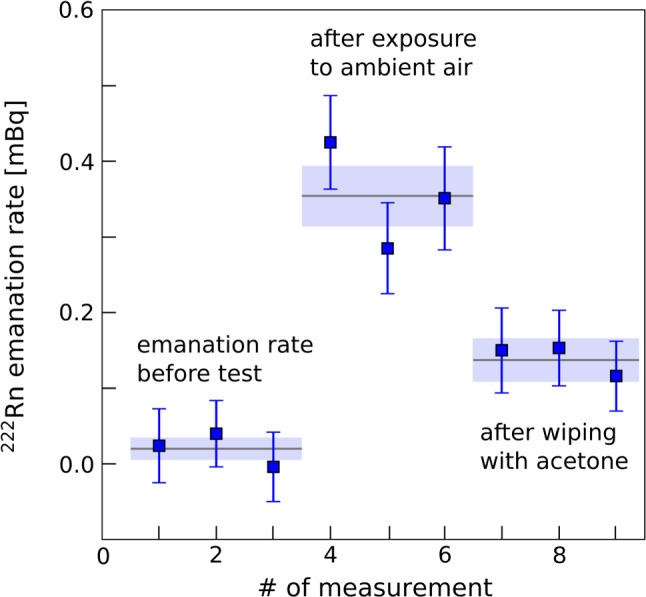
Calculated radon emanation rate after background subtraction of a 3 m$^2$ large PTFE foil before and after a 105 d exposure to ambient air with a radon concentration of $130\,$Bq/m$^3$. The initial emanation rate was not reached again after wiping the sample with acetone-soaked cleanroom wipes.

The second sample was placed in a room with a radon concentration in ambient air of about $130\,$Bq/m$^3$, a factor 3.6 higher with respect to the XENONnT experimental site. The determined $^{222}$Rn emanation rate increase was about the same factor higher and was determined to be 1.05(14) $\upmu$Bq/d/m$^2$. The aerosol concentration in ambient air, which might also impact the plate-out rate, was about $10^6$ particles/m$^3$ (diameter $>0.5$ $\upmu$m) for the second sample. After measuring the radon emanation rate the second sample was cleaned with acetone-soaked cleanroom wipes, which reduced the emanation rate by $\sim 60$%, but still well above the initial value (see Fig. 5). The process of $^{226}$Ra plate-out needs further investigation. Dust from ambient air is thought to be the main source but comprehensive studies on the plate-out mechanisms do not yet exist.

For plate-out protection, PTFE parts, PMTs and electrodes, were stored in nitrogen flushed boxes or were sealed in bags made of mylar foil. For the latter, their radon tightness could be proven by sealing a sample of >30 mBq radon emanation rate in such a mylar foil and placing the bag in a vessel filled with helium carrier gas. After one week, the carrier gas was analysed as it was done for ordinary radon emanation measurements. No significant amount of radon was detected. Mylar bags were also used during the transport of the TPC from the assembly site in the AG-CR to its final destination in the UG-CR, as well as during the installation phase at the experiment site.

## Summary and conclusions

To achieve the target background level in XENONnT, an extensive screening campaign was performed to select construction material with low intrinsic radioactivity. Their gamma emission was measured with high-purity germanium detectors with sensitivities down to $\sim 10$ $\upmu$Bq/kg. ICP-MS was employed for complementary measurements of the $^{238}$U and $^{232}$Th concentrations in the materials. The isotopic activities measured in the radioassay program informed the simulated sensitivity estimates for XENONnT for low-energy nuclear recoils published in [6]. Relative to XENON1T, the selection of materials, as described here, combined with the increased efficacy of fiducialization lead to a reduction ($\sim$17%) in the ER background contribution from detector components, contributing 25(3) events per tonne-year in XENONnT. This contribution is subdominant to $^{222}$Rn and solar neutrinos, contributing with 55(6) and 34(1) events respectively (assuming 1 $\upmu$Bq/kg for $^{222}$Rn). Similarly, the NR background from detector components, 0.32(0.16) events per tonne-year, was cut in half compared to XENON1T. The most significant contributors are the SS cryostat (36%), PMTs (33%), and PTFE components (26%). The neutron veto, which is expected to tag radiogenic events with $\sim$87% efficiency, should further mitigate the materials-induced background to $0.04\,(0.02)$ events per tonne-year. Consequently, radiogenic neutrons would no longer be the dominant source of NR events. The solar $^8$B CEvNS events instead should constitute the largest population of NR background events for dark matter searches, reflecting notable improvements in sensitivity, while also providing their own novel channel of investigation.

As the radioactive noble gas $^{222}$Rn is expected to be the dominant ER background source in XENONnT, the materials have been screened also for their radon emanation rate. Furthermore, the $^{222}$Rn emanation rate of entire detector subsystems was measured during the assembly of XENONnT. Based on these measurements, the locations of the main radon sources could be identified. This knowledge will be used to optimize the efficiency of a novel radon removal system based on cryogenic distillation. The largest $^{222}$Rn source in the XENONnT experiment is the emanation from its cryogenic system. The TPC, with a total activity of 9.3 (3.8)*stat* ($^{+1.2}_{-4.6}$) *sys*  mBq, is the second largest source of radon. The total emanation in XENONnT was estimated to be 35.7 ($^{+4.5}_{-5.9}$) mBq. Assuming a homogeneous distribution within the 8.4 tonnes of xenon in the detector, a final radon activity concentration of 4.2 ($^{+0.5}_{-0.7}$) $\upmu$Bq/kg in the LXe target is expected, a factor of three lower than in XENON1T. XENONnT’s novel radon distillation system will further reduce the radon concentration in LXe, allowing us to achieve the target activity of 1 $\upmu$Bq/kg. Imminent XENONnT TPC data will validate this post-distillation radon concentration projection.

Special emphasis was placed on the cleaning and proper storage of all detector materials during the assembly of XENONnT. Dedicated cleaning procedures were defined for different materials in order to remove dust and lubricants introduced during the material production process. The cleaning agents were selected according to results obtained from gamma-ray screening. For some materials, an increased radon emanation rate was detected after their degreasing treatment, which was associated with a relatively high radium concentration in the cleaning agent. Surface treatments are also important to mitigate background from long-lived radon daughters. For PTFE surfaces, the most critical component in this regard for XENONnT, a reduction up to a factor of six was established for the procedure described above. In order to avoid re-contamination of detector parts, a dedicated cleanroom infrastructure was built for material cleaning, storage, and detector assembly.

## Data Availability

This manuscript has no associated data or the data will not be deposited. [Authors’ comment: The datasets generated during and/or analysed during the current study are available in the repository ‘Supplemental data for material radiopurity control in the XENONnT experiment’, https://zenodo.org/record/5767294.]

## Appendix

See Tables 2, 3, 4, 5 and 6.

**Table 2:** Measured activities of radioactive isotopes in the selected cryostat and TPC materials for XENONnT as well as the cleaning agents used during assembly. Measured values are given with $\pm 1\sigma$ uncertainties, and upper limits are set at 95% C.L. The facility, sample mass, and lifetime are provided for each measurement (as applicable). Samples 18–20 are the averaged results from measurements of different sets of 10-15 PMTs fitting into the germanium spectrometer at once

Sample	Component	Manufacturer	Facility	Mass [kg]	Livetime [d]	Units	$^{238}$U	$^{235}$U	$^{226}$Ra	$^{228}$Ra ($^{232}$Th)	$^{228}$Th	$^{40}$K	$^{60}$Co	$^{137}$Cs
Stainless Steel (304)														
0	Bell/Vessel	Nironit	GeMPI	7.8	11.7	mBq/kg	13(7)	0.7(3)	0.3(1)	0.6(2)	0.5(1)	1.6(6)	2.4(2)	$<0.2$
0	Bell/Vessel	Nironit	ICP-MS	–	–	mBq/kg	3.7(6)	–	–	0.10(8)	–	–	–	–
1	Bell/Vessel	Nironit	GeMPI	7.8	57.1	mBq/kg	4(2)	$0.2(1)^{*}$	1.3(1)	0.9(1)	0.57(6)	1.4(2)	0.61(5)	0.03(2)
1	Bell/Vessel	Nironit	ICP-MS	–	–	mBq/kg	8.6(4)	–	–	$<8.1$	–	–	–	–
2	Bell/Vessel/Electrodes	Nironit	GeMPI	8.4	27.5	mBq/kg	$<11$	$<0.6$	0.6(1)	0.4(1)	0.4(1)	$<2.4$	0.4(1)	$<0.2$
2	Bell/Vessel/Electrodes	Nironit	ICP-MS	–	–	mBq/kg	2.5(3)	–	–	0.4(2)	–	–	–	–
3	Welding Rods (Vessel)	Nironit	GeMPI	2.6	30.6	mBq/kg	$<5.7$	$<0.3^{*}$	3.1(3)	2.9(4)	11.4(7)	7(1)	1.6(2)	$<0.3$
Oxygen-free high-conductivity copper														
4	Field Shaping Rings	Luvata	Gator	71.7	32.5	mBq/kg	$<0.33$	$<0.02$	$<0.18$	$<0.22$	0.18(5)	0.45(14)	0.03(1)	$<0.05$
4	Field Shaping Rings	Luvata	ICP-MS	–	–	mBq/kg	0.03(1)	–	–	0.010(4)	–	–	–	–
5	Guard Rings	Niemet	GeMPI	56.5	42.1	mBq/kg	$<1.6$	$<0.14$	0.13(3)	$<0.06$	$<0.04$	0.6(2)	0.05(1)	$<0.03$
6	Wires	-	GeMSE	12	–	mBq/kg	$<2.3$	–	$<0.1$	$<0.06$	$<0.04$	0.55(2)	0.43(3)	$<0.04$
7	Array Support Plate	Niemet	GeMSE	93.4	35.6	mBq/kg	$<1.06$	–	$<0.21$	$<0.08$	$<0.01$	$<0.42$	0.08(1)	$<0.011$
7	Array Support Plate	Niemet	ICP-MS	–	–	mBq/kg	0.0014(4)	–	–	0.004(1)	–	–	–	–
8	Array Support Pillar	Luvata	GeMPI	57.3	26.2	mBq/kg	$<2.7$	$<0.23$	$<0.06$	$<0.08$	$<0.04$	$<0.27$	0.10(2)	$<0.05$
Plastics														
9	PTFE Reflectors	Amsler and Frey	GeMPI	15.4	25.0	mBq/kg	$<2.4$	$<0.08$	$<0.03$	0.11(4)	$<0.09$	8(1)	–	$<0.07$
9	PTFE Reflectors	Amsler and Frey	ICP-MS	–	–	mBq/kg	$<0.06$	–	–	0.05(2)	–	–	–	–
10	PTFE Reflectors	Amsler and Frey	GeMPI	25.0	19.7	mBq/kg	$<1.0$	$<0.07$	0.15(3)	$<0.1$	$<0.08$	0.08(3)	–	$<0.05$
10	PTFE Reflectors	Amsler and Frey	ICP-MS	–	–	mBq/kg	0.15(7)	–	–	0.03(2)	–	–	–	–
11	PTFE Pillars	Amsler and Frey	GeMPI	15.1	45.0	mBq/kg	$<0.8$	$<0.05$	0.04(1)	$<0.06$	$<0.04$	$<0.42$	–	$<0.01$
11	PTFE Pillars	Amsler and Frey	ICP-MS	–	–	mBq/kg	0.26(9)	–	–	0.10(2)	–	–	–	–
12	PTFE PMT Holders	Amsler and Frey	GeMSE	18.2	19.9	mBq/kg	$<1.9$	–	$<0.1$	$<0.08$	$<0.04$	$<1.0$	$<0.05$	$<0.03$
13	PTFE PMT Holders	Amsler and Frey	ICP-MS	–	–	mBq/kg	$<0.1$	–	–	$<0.04$	–	–	–	–
14	Torlon Reflectors	Drake Plastics	ICP-MS	–	–	mBq/kg	1.8(5)	–	–	0.2(1)	–	–	–	–
15	Torlon Reflectors	Drake Plastics	ICP-MS	–	–	mBq/kg	2.2(6)	–	–	0.4(1)	–	–	–	–
16	PEEK Array Spacers	Spalinger	ICP-MS	–	–	mBq/kg	0.4(1)	–	–	0.12(3)	–	–	–	–
17	PEEK Screws	Solidspot	GeMPI	0.27	23.1	mBq/kg	$<20$	$<1.4$	10(1)	7(1)	6(1)	30(10)	–	$<0.8$
Photosensors and components														
18	R11410 PMTs (average 180 PMTs)	Hamamatsu	Gator	–	–	mBq/PMT	9(2)	0.4(1)	0.47(2)	0.47(7)	0.46(2)	14.2(5)	1.05(3)	$<0.14$
19	R11410 PMTs (average 60 PMTs)	Hamamatsu	GeMPI	–	–	mBq/PMT	14(7)	0.5(1)	0.52(4)	0.6(1)	0.45(5)	18.6(9)	1.27(6)	$<0.13$
20	R11410 PMTs (average 99 PMTs)	Hamamatsu	GeMSE	–	–	mBq/PMT	6.5(3)	–	0.32(4)	0.33(5)	0.19(1)	11.1(4)	0.71(3)	$<0.06$
21	Ceramic Stem	Hamamatsu	GeMPI	1.5	20.7	mBq/kg	2.7(5)	0.13(2)	0.29(2)	0.17(2)	0.12(1)	2.7(3)	$<0.003$	$<0.009$
22	Ceramic Stem	Hamamatsu	GeMPI	1.6	22.8	mBq/kg	3.4(5)	0.12(2)	0.22(1)	0.20(2)	0.07(1)	0.13(2)	$<0.002$	$<0.01$
23	Bases/components	Fralock/various	GeMSE	1.9	7.0	mBq/piece	1.5(1)	–	0.7(3)	0.14(1)	0.053(3)	0.29(5)	$<0.003$	$<0.002$
24	HV Connectors/PTFE/Copper	Accu-Glass/custom	GeMSE	1.0	27.5	$\upmu$Bq/piece	$<310$	–	$<37$	$<42$	$<14$	140(90)	50(10)	$<3.3$
25	Coaxial Cables	Huber and Suhner	GeMPI	1.5	22.6	mBq/kg	$<74$	$<1.4$	2.4(5)	$<1.5$	1.4(5)	40(8)	$<0.2$	$<0.6$
26	Kapton Cables	Accu-glass	GeMPI	0.1	29.3	mBq/kg	$<56$	$<11$	8(2)	$<9.0$	$<3.4$	110(30)	$<3.3$	$<4.2$
27	Optical Fiber	Ratioplast	ICP-MS	–	–	mBq/kg	7(3)	–	–	7(2)	–	340(90)	–	–
Miscellaneous														
28	M5 Screws (Ag), TPC	U-C Components	GeMPI	0.5	27.6	mBq/kg	$<43$	$<1.4$	$<1.1$	3(1)	3.3(6)	12(3)	53(4)	$<0.6$
29	M6 Screws (Ag), TPC	U-C Components	GeMPI	1.0	23.9	mBq/kg	$<15$	$<3.4$	$<1.6$	$<3.0$	2.7(7)	18(5)	6.7(7)	$<0.9$
30	M8 Screws (Ag), TPC	U-C Components	GeMPI	0.9	22.8	mBq/kg	50(20)	$<2.1$	2.0(5)	3.4(8)	3.9(6)	13(3)	52(4)	$<0.8$
31	M8 Screws (Ag), Bell	ALCA	GeMPI	1.2	21.6	mBq/kg	$<29$	$<0.8$	0.8(3)	2.0(5)	7.6(6)	5(2)	4.9(4)	$<0.3$
32	SMD Resistors	OHMITE	GeDSG	0.01	6.9	$\upmu$Bq/piece	110(50)	2.3(5)	29(2)	13(2)	15(1)	60(10)	$<1.1$	$<0.6$
Cleaning solutions														
33	HARO Clean 100	HAROSOL	Corrado	1.0	9.8	mBq/kg	$<710$	$<20$	11(6)	$<18$	$<21$	4600(300)	$<8$	$<7$
34	HARO Clean 188	HAROSOL	Corrado	1.5	6.0	mBq/kg	$<24400$	–	$<35$	520(310)	$<18$	$5.8(4)\times 10^6$	$<175$	$<133$
35	HARO Clean 106	HAROSOL	Corrado	0.6	3.1	mBq/kg	$2.1(1)\times 10^3$	–	$<20$	$<77$	$<16$	750(160)	$<9$	$<15$
36	P3-Almeco 36	Henkel	Bruno	0.1	3.9	mBq/kg	$14(3)\times 10^3$	790(160)	98(27)	$<91$	$<113$	$15(2)\times 10^3$	$<19$	$<21$
37	Tickopur R33	Dr. H. Stamm	Giove	1.0	5.0	mBq/kg	$<7400$	–	36(13)	$<429$	$<11$	$1.55(9)\times 10^6$	$<49$	$<65$
38	Elma clean 65	Elma	Giove	1.0	7.1	mBq/kg	430(200)	–	6(3)	$<12$	$<8$	1190(90)	$<1.6$	$<4$
39	Elma clean 70	Elma	Giove	1.1	2.7	mBq/kg	$<17100$	–	53(18)	$<340$	19(9)	$1.45(9)\times 10^6$	$<65$	$<156$
40	$\hbox {HNO}_3$ (69%)	Roth	Corrado	0.5	5.1	mBq/kg	$<970$	–	$<19$	$<22$	$<27$	$<80$	$<4$	$<7$

**Table 3:** Measured activities of radioactive isotopes in the neutron veto, purification, and calibration subsystems. Measured values are given with $\pm 1\sigma$ uncertainties, and upper limits are set at 95% C.L. The facility, sample mass, and lifetime are provided for each measurement (as applicable)

Sample	Component	Manufacturer	Facility	Mass [kg]	Livetime [d]	Units	$^{238}$U	$^{235}$U	$^{226}$Ra	$^{228}$Ra ($^{232}$Th)	$^{228}$Th	$^{40}$K	$^{60}$Co	$^{137}$Cs
Neutron Veto														
41	R5912 PMT Body	Hamamatsu	GeMPI	0.2	6.9	mBq/kg	70(30)	$<6.4$	52(4)	37(4)	30(3)	360(40)	$<1.8$	$<1.3$
42	R5912 PMT low radioactivity Glass	Hamamatsu	GeCris	0.4	3.9	mBq/kg	700(200)	40(10)	700(30)	740(50)	670(45)	1000(100)	$<3.0$	$<9.7$
43	Polyethylene PMT Holders	Plastotecnica emiliana	GeMPI	0.1	13.8	mBq/kg	$<19$	$<2.2$	2.1(8)	$<2.2$	$<2.1$	$<22$	–	$<0.6$
44	SS Support Structure	Galli and Morelli	GeMPI	0.5	27.5	mBq/kg	$<25$	$<0.55$	0.8(2)	1.1(4)	2.3(4)	$<4.9$	4.1(4)	$<0.2$
45	ePTFE Reflectors	Applitecno Service	ICP-MS	–	–	mBq/kg	0.3(1)	–	–	0.12(4)	–	–	–	–
46	Gadolinium Sulfate	NYC	GeMPI	1.0	20.7	mBq/kg	$<14$	$<0.5$	0.9(2)	0.4(2)	1.2(2)	$<4.1$	–	$<0.06$
47	Gadolinium Sulfate	Treibacher	GeMPI	1.0	6.6	mBq/kg	$<43$	$<3.6$	3.4(7)	23(2)	190(10)	9(4)	–	$<0.8$
Purifying Getter Materials														
48	LXe Filter 2$_{a}$	BASF	Corrado	0.3	23.8	mBq/kg	3200(700)	–	145(10)	70(20)	86(9)	920(90)	$<6$	$<7$
49	LXe Filter 2$_{b}$	BASF	Corrado	0.4	10.4	mBq/kg	2900(760)	–	150(10)	70(20)	84(16)	720(90)	$<6$	$<6$
50	LXe Filter 2$_{c}$	BASF	Corrado	0.1	7.2	mBq/kg	3600(1300)	–	1050(40)	460(60)	560(50)	1500(200)	$<11$	$<8$
Calibration														
51	Polyurethane Belt	BRECOFlex	GeMPI	0.6	9.5	mBq/kg	46(19)	$<2.5$	13(1)	5(1)	4(1)	93(17)	1.9(6)	$<0.7$
52	Source Box	–	Giove	7.3	13.8	mBq/kg	$<26.4$	$<1.7$	$<0.9$	1.0(6)	1.7(4)	$<1.2$	6.2(4)	$<0.2$
53	Source Box Clamp	McMaster	Corrado	0.9	24.5	mBq/kg	$<395$	$<44$	46(4)	$<15.5$	15(5)	$<32$	12(2)	$<2$
54	SS316 Tube	Swagelok	GSOr	2.9	7.1	mBq/kg	$<33$	$<2.8$	5.3(7)	6.2(9)	14(1)	$<7.5$	2.5(3)	$<0.8$
55	SS304 Beam Pipe	Weizmann	GeMPI	3.1	16.7	mBq/kg	$<63$	$<1.8$	1.7(5)	6(1)	6(1)	$<10$	6.8(7)	$<0.5$
56	SS304 Support Pipe	Weizmann	GeMPI	0.5	27.6	mBq/kg	36(18)	$<0.9$	0.6(3)	1.9(8)	6.8(8)	$<15$	6.5(8)	$<0.9$
57	SS316 Clamps	McMaster	GeMPI	2.2	5.6	mBq/kg	150(60)	$<8.8$	102(5)	19(2)	18(2)	7(3)	12(1)	–
58	SS304 Bellow	MDC	GeMPI	3.5	13.7	mBq/kg	$<47$	$<1.3$	2.0(4)	5.3(9)	9.8(9)	$<3.6$	7.1(7)	$<0.5$
59	SS304 Flanges	MDC	GeMPI	12.0	20.6	mBq/kg	24(12)	1.0(4)	0.4(1)	1.6(4)	5.0(4)	1.6(6)	11.2(8)	$<0.2$
60	SS304 U-Tube	CG	GeMPI	0.6	16.2	mBq/kg	$<150$	$<5.8$	$<1.9$	$<6.6$	4(1)	15(8)	45(4)	$<1.5$

**Table 4:** Measured activities of radioactive isotopes in some materials that were rejected. Measured values are given with $\pm 1\sigma$ uncertainties, and upper limits are set at 95% C.L. The facility, sample mass, and lifetime are provided for each measurement (as applicable)

Sample	Component	Manufacturer	Facility	Mass [kg]	Livetime [d]	Units	$^{238}$U	$^{235}$U	$^{226}$Ra	$^{228}$Ra ($^{232}$Th)	$^{228}$Th	$^{40}$K	$^{60}$Co	$^{137}$Cs
R0	SS304	Nironit	GeMPI	8.8	17.5	mBq/kg	$<21$	$<0.8$	1.7(2)	1.1(3)	1.2(2)	$<3.0$	0.8(1)	$<0.2$
R0	SS304	Nironit	ICP-MS	–	–	mBq/kg	3.7(5)	–	–	0.5(1)	–	–	–	–
R1	SS20	Terni	GeMPI	10.3	12.3	mBq/kg	$<24$	$<1.2$	$<69$	2.1(4)	7.1(6)	1.3(6)	14(1)	$<0.2$
R1	SS20	Terni	ICP-MS	–	–	mBq/kg	4.5(8)	–	–	5.0(8)	–	–	–	–
R2	SS27	Terni	GeMPI	7.2	16.6	mBq/kg	$<40$	$<1.4$	$<0.6$	$<1.1$	2.1(4)	$<1.4$	23(2)	$<0.2$
R2	SS27	Terni	ICP-MS	–	–	mBq/kg	15(3)	–	–	1.4(2)	–	–	–	–
R3	SS304	Nironit	GeMPI	8.0	13.8	mBq/kg	$<11$	$<0.9$	2.0(2)	2.2(3)	5.4(4)	1.7(5)	0.31(5)	$<0.1$
R3	SS304	Nironit	ICP-MS	–	–	mBq/kg	3.5(6)	–	–	3(1)	–	–	–	–

**Table 5:** $^{222}$Rn emanation rate investigation of materials and components from XENONnT subsystems. Columns indicate the sample ID; sample name, manufacturer and description; and the measurement facility used: proportional counter (PC). Mass and/or length are provided when applicable. Measured values are given with $\pm 1\sigma$ uncertainties and upper limits are set at 90% C.L

Sample	Component	Manufacturer	Description	Facility	Mass [kg]	Length [m]	Units	$^{222}$Rn	Used in XENONnT	
									In Cryostat [m]	In Cable Pipe 2 [m]
Cables in CRY										
Rn1	Accuglass-1	Reliable Hermetic Seals	Accuglass 100670 single-core kapton wire	PC	0.071	100	mBq/m	$\le$0.28 $\times$ 10$^{-3}$	0	0
Rn2	Accuglass-2	Reliable Hermetic Seals	Accuglass 100670 single-core kapton wire	PC	0.070	100	mBq/m	0.55 (19) $\times$ 10$^{-3}$	0	998
Rn3	Accuglass-3	Reliable Hermetic Seals	Accuglass 100670 single-core kapton wire	PC	–	200	mBq/m	0.41 (15)$\times 10^{-3}$	1821	0
Rn4	Habia RG196A/U	Koax24	PTFE coaxial cable RG196	PC	0.999	100	mBq/m	12 (2) $\times$ 10$^{-3}$	0	0
Rn5	H+S 1	Novitronic AG	Huber+Suhner RG196 A/U	PC	–	50	mBq/m	< 0.48 $\times$ 10$^{-3}$	192	442
Rn6	H+S 2	Huber+Suhner	Huber+Suhner RG196 A/U	PC	–	100	mBq/m	0.38 (22) $\times$ 10$^{-3}$	991	0
Rn7	H+S 3	Huber+Suhner	Huber+Suhner RG196 A/U	PC	–	50	mBq/m	< 1.36 $\times$ 10$^{-3}$	264	614
Rn8	Pasternack	Pasternack E.	Pasternack RG196 A/U	PC	–	198	mBq/m	0.48 (18) $\times$ 10$^{-3}$	0	0
Plastics in Rn-DST										
Rn9	UHMWPE	–	Gasket material	PC	0.160	–	mBq	0.09 (2)	Mag-Pump 4	
Rn10	Iglidur A180	Igus	Gasket material white	PC	0.178	–	mBq	$\le$ 0.05	Mag-Pump 3	
Rn11	Iglidur J	Igus	Gasket material yellow	PC	0.266	–	mBq	$\le$ 0.02	Mag-Pump 1 and 2	
Rn12	Iglidur A350	Igus	Gasket material blue	PC	0.145	–	mBq	0.06 (3)	NO	
LXe pump and filter in LXe-PUR										
Rn13	Viton O-ring	Barber Nichols	.103$\times$4.487 fitting	PC	0.004	–	mBq	0.76 (8)	YES	
Rn14	SS cryogenic valve	Thermomess	Bellows-sealed globe valve (after installation)	PC	–	–	mBq/valve	0.22 (4)	YES	
Rn15	SS cryogenic valve	Thermomess	Bellows-sealed globe valve	PC	2.713	–	mBq/valve	0.11 (4)	YES	
Rn16	Aluminum rotor	Barber Nichols	Alumina rotor from LXe-Pump	PC	0.062	0.06	mBq	< 0.017	YES	
Rn17	LXe Filter 2$_{a}$	BASF	Sample 48 in Table 3	PC	0.3	–	mBq/kg	50 (2)	NO	
Rn18	LXe Filter 2$_{c}$	BASF	Sample 50 in Table 3	PC	0.271	–	mBq/kg	369 (15)	NO	

**Table 6:** $^{222}$Rn emanation rate from xenon handling systems and the TPC of XENONnT. Columns indicate the measurement ID; sample name, manufacturer and description; and the measurement facility used: proportional counter or radon monitor (PC or RM). Previous results of XENON1T are further referenced with their ID in [10]. Measured values are given with $\pm 1\sigma$ uncertainties and upper limits are set at 90% C.L

Sample	Component	Description and/or manufacturer	Facility	$^{222}$Rn [mBq]
CRY				
Rn19	Cryostat	Costruzioni Generalli	PC	1.9 (2)
Rn20	Cooling Tower, Cryostat Pipe, HE and Heater,	retained from XENON1T	RM	17 (3)
	Cable Feedthrough 1 and Cable Pipe 1			
Rn21	Cable Feedthrough 2	–	PC	1.6 (2)
Rn22	Cable Pipe 2	–	PC	0.9 (1)
–	**Integral CRY**	–	–	**21 (3)**
GXe-PUR				
#23 in [10]	Mag-pump 1	Muenster U.	–	0.3 (1)
#16 in [10]	GXe Filter 1	SAES ($\sim$4 kg)	–	1.2 (2)
#17 in [10]	GXe Filter 2	SAES ($\sim$4 kg)	–	0.24 (3)
–	**Integral GXe-PUR**	–	–	**1.7 (2)**
LXe-PUR				
Rn23	Vacuum insulated pipe	Costruzioni Generalli	PC	0.22 (5)
Rn24	Purity Monitor	U. Tokyo	PC	0.25 (6)
Rn25	LXe Filter 1	SAES St707 ($\sim$3 kg)	PC	0.24 (3)
Rn26	Heat exchanger	DATE, 500W Xe/$\hbox {LN}_{2}$	PC	1.3 (1)
Rn27	LXe-Pump	Barber Nichols	PC	1.6 (2)
–	**Integral LXe-PUR**	–	–	**3.6 (2)**
Rn-DST				
Rn28	Distillation column	–	PC	1.7 (2)
#20$_{\text {a}}$ in [10]	Q-Drive	–	–	2.5 (1)
Rn29	GXe Filter 3	SAES ($\sim$0.5 kg)	Estimated from #18 in [10]	0.09 (3)
Rn30	4 Mag-pumps	–	PC	0.30 (5)
Rn31	Mag-Pumps inlet buffer	U. Muenster	estimated from [10, 30]	0.24 (12)
Rn32	Mag-Pumps outlet buffer	Hositrad	PC	0.94 (9)
Rn33	Xe–Xe heat exchanger	Secespol	PC	< 0.03
Rn34	Xe condenser	U. Muenster	estimated from [10, 30]	0.07 (4)
–	**Integral Rn-DST (Rn30-Rn34)**	–	–	1.6 (2)
TPC				
Rn35	**Integral TPC**	–	RM	9.3 (3.8)*stat* ($^{+1.2}_{-4.6}$) *sys*

## Footnotes

1 http://www.barber-nichols.com.

2 https://www.chartindustries.com/.

3 Note that upper limits in Sect. 3, were given at 95% C.L., in order to be consistent with previous XENON1T gamma-screening [9] and radon-emanation [10] references.

4 https://harosol.com/en/.

5 http://www.henkel-adhesives.com.

6 http://www.elma-ultrasonic.com.

7 http://www.xia.com/ultralo.html.
